# Control of Intracellular *Francisella tularensis* by Different Cell Types and the Role of Nitric Oxide

**DOI:** 10.1155/2014/694717

**Published:** 2014-07-21

**Authors:** Sarah L. Newstead, Amanda J. Gates, M. Gillian Hartley, Caroline A. Rowland, E. Diane Williamson, Roman A. Lukaszewski

**Affiliations:** Dstl, Porton Down, Salisbury SP4 0JQ, UK

## Abstract

Reactive nitrogen is critical for the clearance of *Francisella tularensis* infections. Here we assess the role of nitric oxide in control of intracellular infections in two murine macrophage cell lines of different provenance: the alveolar macrophage cell line, MH-S, and the widely used peritoneal macrophage cell line, J774A.1. Cells were infected with the highly virulent Schu S4 strain or with the avirulent live vaccine strain (LVS) with and without stimuli. Compared to MH-S cells, J774A.1 cells were unresponsive to stimulation and were able to control the intracellular replication of LVS bacteria, but not of Schu S4. In MH-S cells, Schu S4 demonstrated control over cellular NO production. Despite this, MH-S cells stimulated with LPS or LPS and IFN-*γ* were able to control intracellular Schu S4 numbers. However, only stimulation with LPS induced significant cellular NO production. Combined stimulation with LPS and IFN-*γ* produced a significant reduction in intracellular bacteria that occurred whether high levels of NO were produced or not, indicating that NO secretion is not the only defensive cellular mechanism operating in virulent *Francisella* infections. Understanding how *F. tularensis* interacts with host macrophages will help in the rational design of new and effective therapies.

## 1. Introduction

*Francisella tularensis* is a Gram-negative, facultative intracellular bacterium, which is the causative organism of the disease tularemia [1]. There are two main biovars of* F. tularensis* which cause disease in humans:* F. tularensis* subsp.* tularensis*, which is highly virulent and potentially fatal (designated type A), and the less virulent* F. tularensis* subsp.* holartica *(designated type B), a mutation of which has resulted in further attenuation and its development as a live attenuated vaccine, the live vaccine strain (LVS). In parts of the world (Scandinavia, North America, and parts of Asia)* F. tularensis* is harboured by the local wildlife, for example, rabbits or deer, that can transmit the bacterium to humans [2].

Protection against an inhaled infection with* F. tularensis* is highly desired, as it is estimated that as little as 25 colony-forming units (cfu) can cause fatal disease [3]. Currently, there is no licensed vaccine for tularemia and antibiotics have limited efficacy due to the infection being intracellular in nature and somewhat difficult to diagnose [4]. Protection against inhalational exposure with* F. tularensis* Schu S4 would be facilitated by further understanding of the mechanisms of resistance operating in the respiratory tract and the lungs. As alveolar macrophages reside in the lungs, they provide a first line of defence against an aerosol infection and, to date, infection of these cells with* F. tularensis* has not been extensively studied.

MH-S cells are a murine alveolar macrophage cell line, created by obtaining cells from a bronchoalveolar lavage, which were then transformed with simian virus 40 (SV40) to produce a rapidly proliferating cell line [5]. J774A.1 cells are a well-defined and widely used murine peritoneal macrophage cell line. Both these macrophage cell types can support the growth of intracellular pathogens such as* F. tularensis, Mycobacterium tuberculosis* [6], and* Legionella pneumophila *[7] and both can secrete cytokines and nitric oxide [8]. Here, we have compared the cellular responses of these two macrophage cell lines, J774A.1 and MH-S, to infection with* F. tularensis*.

Selected isolated components of bacteria such as peptidoglycan, lipopolysaccharide, synthetic CpG, and proinflammatory cytokines have all been used to study activation and the protective responses of macrophages* in vitro* [9]. One of the known macrophage resistance mechanisms against* F. tularensis* is the induction of nitric oxide synthase (iNOS) and NO secretion [10–12]. NO is a short-lived inorganic free radical gas derived from L-arginine by NOS activity [13], which has an antimicrobial effect important in the innate immune system.

The observed ability of more virulent* F. tularensis *strains to survive within macrophages and other cells may depend on their capacity to suppress such antibacterial activities of the host cells [14]. It has been previously reported that type A strains of* F. tularensis* possess the enzyme citrulline ureidase (ctu) [15], which recently has been described as a virulence factor, enabling the bacteria to limit the amount of arginine available to the host cell and thereby restrict the production of reactive nitrogen [16]. A Δctu mutant of* F. tularensis* Schu S4 was significantly attenuated in mice and, when used to infect macrophages* in vitro,* was more susceptible to killing due to the observed enhanced levels of nitrite production (measured as the stable oxidative product of NO and an indicator of NO production), compared with Schu S4-infected macrophages [16]. These findings led us to question whether NO production is effective in countering the virulence of the Schu S4 strain and whether it is the only effective mechanism available to host cells.

We have tested the ability of combinations of stimulants to induce significant NO synthesis in the J774A.1 and MH-S cell lines. We have also used the chemical inhibitor of NO synthesis, N^G^-monomethyl-L-arginine, to investigate the specific influence of NO induction on the resistance of mammalian cells to infection with tularemia strains of differing virulence* in vivo*. We have also assessed the effect of the induced NO on the intracellular growth of* F. tularensis *in each cell line to determine whether any observed difference in functionality can be correlated with cellular source.

## 2. Materials and Methods

### 2.1. Bacteria

*F. tularensis* LVS was derived directly from an original NDBR 101, lot 4 vaccine ampoule produced during the 1960s. Prior to reconstitution, vaccine ampoules were stored at −20°C.* F. tularensis* Schu S4 was originally isolated from a human case of tularemia in 1941 and has been passaged through animals.

### 2.2. Cell Lines

MH-S alveolar macrophages and J774A.1 peritoneal macrophages (ECACC, PHE, Porton Down, UK) were cultured in RPMI1640 (plus 10% FCS and 2% L-glutamine) or DMEM (10% FCS and 2% L-glutamine), respectively (all from Invitrogen Ltd, Paisley, UK). Both cell lines were cultivated in 5% CO_2_ at 37°C in a humidified environment. Cells were seeded into 24-well plates (Corning) at a density of 5 × 10^5^ cells/mL and allowed to adhere overnight. Immediately before infection the cells were visually inspected to ensure a confluent monolayer (1 × 10^6^/well).

### 2.3. Stimulation

Confluent monolayers of cells were stimulated with 2.5 *μ*g/mL lipopolysaccharide from* Escherichia coli* (Sigma, Gillingham, UK) or 1 *μ*g/mL recombinant mouse interferon gamma (IFN-*γ*) (R&D systems Europe Ltd, Abingdon, UK) or 10 *μ*g/mL CpG 10109 (Coley Pharmaceuticals, USA) or peptidoglycan (Sigma, Gillingham, UK) at 20 *μ*g/mL or TNF-*α* (AbD Serotec, Kidlington, UK) at 20 *μ*g/mL. These concentrations were selected following optimisation for maximum production of nitric oxide over a period of 24 hr.

### 2.4. Cytokine Release

Cytokine release was measured in the supernatant of uninfected cells following 24 hours of stimulation. The suite of cytokines measured using flow cytometry and mouse inflammation cytometric bead array (CBA) kits (BD Biosciences, UK) was TNF-*α*, IFN-*γ*, IL-6, IL-12, and CCL2. The CBA kits were used in accordance with the manufacturer's instructions and analysed using FACScanto II (BD).

### 2.5. Measuring Nitric Oxide Production and Inhibition

NO concentration was measured as the stable oxidized metabolite and nitrite (NO_2_^−^) using a Griess reaction kit (Promega UK Ltd, Southampton, UK). The limit of detection (LOD) was 2.5 *μ*M (125 pmol). Manufacturer's instructions were followed. Briefly, 50 *μ*L of sample and 50 *μ*L of sulfanilamide solution (1% sulfanilamide in 5% phosphoric acid) were incubated at room temperature and protected from light for 5–10 minutes. Subsequently, 50 *μ*L of 0.1% N-1-naphthylethylenediamine dihydrochloride in water was added, reincubated, and protected from light for a further 5–10 minutes. The absorbance was read at 540 nm and compared to a known standard.

The nitric oxide inhibitor N^G^-monomethyl-L-arginine (L-NMMA) (Sigma, Gillingham, UK) was used at a concentration of 4 mM and added after stimulation and remained for the duration of the assay.

### 2.6. Infection and Stimulation of Cells with Bacteria

All infection experiments were performed under containment level 3 (CL3) conditions (necessary for infection studies with type A strains of* F. tularensis*) with a range of stimulants.

Both strains of* F*.* tularensis* were cultured on blood cysteine glucose agar supplemented with 50% glucose, 10% histine, 10% cysteine, and defibrinated horse blood at 50°C. The multiplicity of infections (MOI) required to achieve comparable levels of infections between the strains and cells was determined in initial experiments. MOIs used in NO experiments were LVS 100 : 1, Schu S4 10 : 1 for MH-S cells, and LVS 10 : 1, Schu S4 1 : 1 for J774A.1 cells. Bacteria and cells were incubated for 30 minutes. Following this all of the supernatant was removed, the cells were not washed, and 10 *μ*g/mL gentamicin (Sigma, Gillingham, UK) was added for 30 minutes to kill any extracellular bacteria. Workup of the method demonstrated that this concentration of gentamicin is sufficient to kill all extracellular* Francisella* of either strain. This was deemed time 0 and stimulants were added. Gas packs (Biomerieux, Basingstoke, UK) were used to supply CO_2_ during the infection and stimulation of the cell lines.

Supernatant from the wells was taken for measurement of nitrite and cytokine production and bacteria were enumerated.

### 2.7. Intracellular Counts

Intracellular bacterial counts were achieved by lysing the macrophages with distilled water and vigorous pipetting for approximately five minutes. Relevant dilutions (made in PBS) were then pipetted out onto BCGA agar and incubated at 37°C for three days before colonies were counted.

### 2.8. Bacterial Sensitivity to NO

Spermine NONOate was used as an NO donor. This compound is stable under alkaline conditions but disassociates releasing free NO at pH 7.4 or below. Increasing concentrations of Spermine NONOate were used to determine if the* Francisella* strains had similar sensitivity to NO under extracellular conditions (PBS room temperature). Spermine NONOate (Cambridge Bioscience, UK) was used according to the manufacturer's instructions.

### 2.9. Statistical Analysis

In order to quantify the NO response of either cell line to stimulation or inhibition, at least 3 independent replicates were used to derive mean values ± standard errors of the mean (SEM). For the bacterial growth assays, the increase in intracellular bacterial counts achieved after 24 hours in unstimulated (media only) cells was taken as 100% and the change in bacterial counts from stimulated cells was expressed as a percentage of this. Under this system, a percentage increase of less than 1% denotes an actual decrease in bacterial numbers from the *t* = 0 initial infection. Thus the data from independent experiments were combined. Student's *t*-test was used to analyse the data and determine significant differences at the levels of *P* < 0.05 (∗), *P* < 0.01 (∗∗), and *P* < 0.001 (∗∗∗).

## 3. Results

### 3.1. Response of Cell Lines to Infection with* F. tularensis*

Before stimulation and infection studies were conducted, the ability of both Schu S4 and LVS to be phagocytosed by and colonise J774A.1 and MH-S cells was compared.

In either cell line, at both time points, Schu S4 colonised cells significantly faster than LVS (*P* < 0.005, Table 1). After 30 mins of infection with a multiplicity of infection (MOI) of 10 : 1 (bacteria to cells) J774A.1 cells contained significantly more bacteria (Schu S4 or LVS) than MH-S cells (*P* < 0.002); however this difference was not significant at the 120-minute time point.

Once infection was established, survival of bacteria within unstimulated macrophages (regardless of cell line) was not significantly different between the two strains, each achieving on average 2 logs of growth over 24 hours of incubation (data not shown).

The MOIs were adjusted in subsequent experiments to compensate for differences in uptake, ensuring comparable infection rates between bacterial strains and cell lines. The starting infection established was in the region of 100 bacteria per 10^6^ cells per mL. The following MOIs were used on MH-S cells: LVS 100 : 1, Schu S4 10 : 1 and on J774A.1 cells: LVS 10 : 1, Schu S4 1 : 1.

### 3.2. Cytokine Production

**Figure 1:**
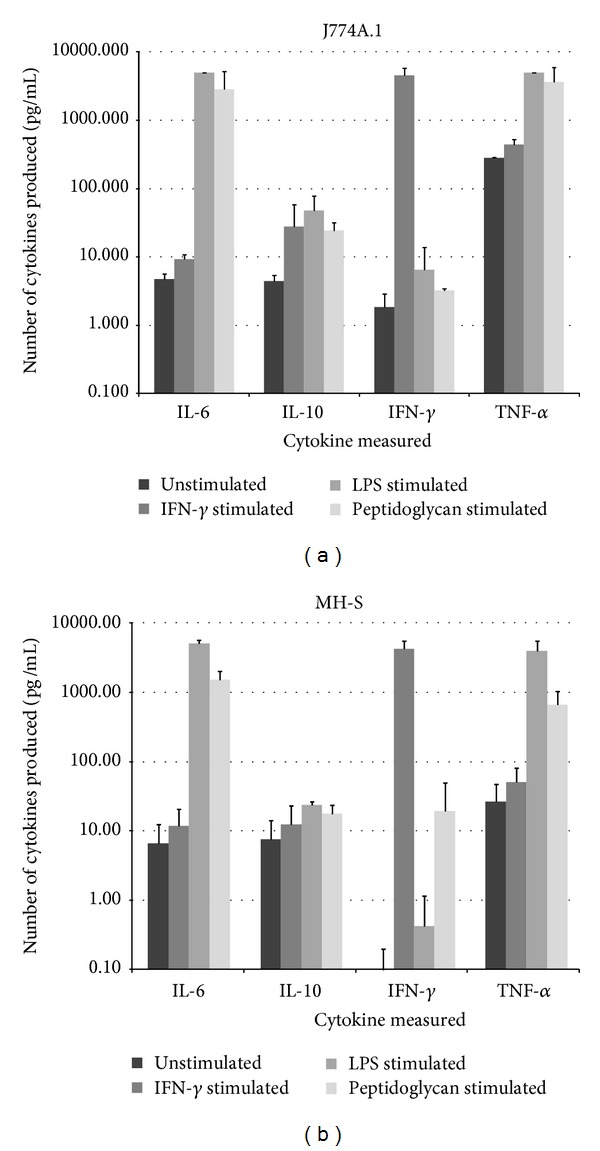
((a) and (b)) Cytokine production from stimulated J774A.1 and MH-S cells. Cytokine concentrations were measured 24 hours after stimulation with LPS (5 *μ*g/mL) or IFN-*γ* (1 *μ*g/mL). Values are the means and SEM from at least three independent experiments. There were significant differences in TNF-*α* production between the cell lines for unstimulated, LPS stimulated and peptidoglycan stimulated cells.

Cytokine production was compared between J774A.1 and MH-S cells. The cytokine profiles of the cells were similar in that low levels of cytokines were detected in unstimulated cells with the exception of CCL2 (MCP-1). Also both cell lines stimulated with LPS (used here as an immunostimulant at supraphysiological levels) induced IL-6 and TNF-*α* production (Figures 1(a) and 1(b)). IFN-*γ* stimulation produced further release of IFN-*γ* and peptidoglycan stimulation produced high levels of IL-6 and TNF-*α* (data not shown). J774A.1 cells produce significantly more TNF-*α* than MH-S cells under all conditions tested except for response to LPS (*P* < 0.005). Combined stimulation with LPS and IFN-*γ* was not tested.

### 3.3. Nitrite Production

**Figure 2:**
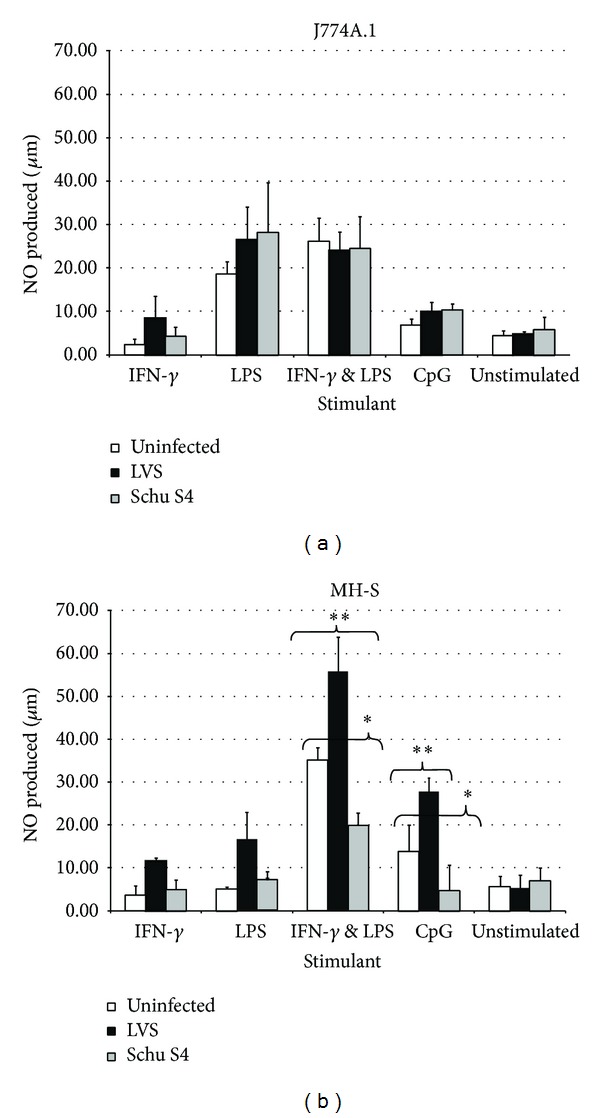
((a) and (b)) Nitrite production from stimulated cells: J774A.1 (a) and MH-S (b). Stimulant concentrations added were IFN-*γ* (1 *μ*g/mL), LPS (5 *μ*g/mL), and IFN-*γ* + LPS (1 *μ*g/mL + 5 *μ*g/mL). Nitrite measurements were taken 24 hours after stimulation and/or infection. Values are the means and SEM from at least three independent experiments. Significant differences in production of nitrite from stimulation or stimulation and infection are marked with asterisks. Significance levels of ∗*P* < 0.05, ∗∗*P* < 0.01, and ∗∗∗*P* < 0.001 by Student's *t*-test.

Production of nitrite from unstimulated cells of either cell line was consistently below 10 *μ*M (media Figure 2). Infection with either LVS or Schu S4 did not cause an increase in either cell line in the production of nitrite.

When stimulated without infection, J774A.1 cells produced NO in response to LPS and IFN-*γ* plus LPS (both *P* < 0.05) but not to IFN-*γ* only or CpG (Figure 2(a)). The MH-S cells did not respond to LPS by the production of NO but produced NO in response to IFN-*γ* plus LPS and to CpG (*P* < 0.05Figure 2(b)).

Infection of J774A.1 with either LVS or Schu S4 did not alter the production of NO above the effect of the stimulant (Figure 2(a)). In contrast infection of the MH-S cell line with LVS leads to increased production of NO which was significant for the IFN-*γ* plus LPS and for the CpG treated cells (*P* < 0.01) (Figure 2(b)). Infection with Schu S4 (Figure 2(b)) had a dramatic effect on the MH-S production of nitrite, reducing it in all stimulated groups and significantly so in cells stimulated with both IFN-*γ* plus LPS and CpG (*P* < 0.05).

### 3.4. Bacterial Sensitivity to Extracellular NO

The LVS and Schu S4 strains were tested for their relative sensitivity to extracellular NO. When tested at concentrations ranging from 0 mM to 2 mM NO in PBS, both strains were sensitive to extracellular NO, with a maximal reduction in viable counts (for both strains) of 1 log over 1 hour of exposure to 2 mM (Table 2).

### 3.5. Intracellular Bacterial Counts of Stimulated Macrophages

**Figure 3:**
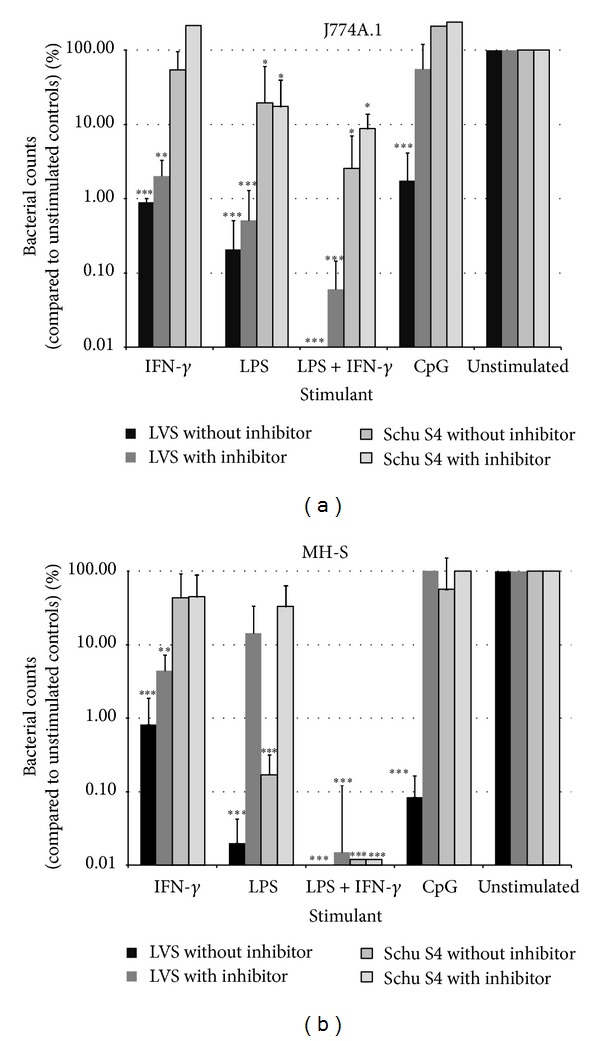
((a) and (b)) Intracellular counts 24 hours after stimulation and infection of J774A.1 cells (a) and MH-S cells (b). Stimulant concentrations added were IFN-*γ* (1 *μ*g/mL), LPS (5 *μ*g/mL), IFN-*γ* + LPS (1 *μ*g/mL + 5 *μ*g/mL), and CpG (10 *μ*g/mL). Nitric oxide production was inhibited by 4 mM N^G^-monomethyl-L-arginine added before stimulation. Values are the means and SEM from at least three independent experiments, with the starting infection given a value of 1% and the maximal growth achieved (in unstimulated cells) as 100%. Significant differences in intracellular counts between stimulated and unstimulated cells are shown (significance ∗*P* < 0.05, ∗∗*P* < 0.01, and ∗∗∗*P* < 0.001 by Student's *t*-test).

Intracellular bacteria were enumerated from cells that were stimulated with CpG, LPS, IFN-*γ* separately, or LPS and IFN-*γ* combined and infected with either LVS or Schu S4 (Figures 3(a) and 3(b)). The results presented in Figure 3 are combined from at least 3 replica experiments by converting the growth achieved by the bacterial strain in unstimulated cells to 100%, with the starting count (*t* = 0) being equal to 1%. Thus the growth achieved by the bacteria in stimulated cells is expressed as a percentage of maximal growth possible: 1% equates to no change from the starting infection and values of less than 1% represent a reduction from the initial level of infection at *t* = 0. The initial infection was generally in the region of 100 bacteria to 1 × 10^6^ cells.

*Intracellular growth of LVS* was significantly reduced in either cell line under all of the stimulating conditions compared to growth in unstimulated cells (*P* < 0.001). This was more pronounced in MH-S cells stimulated with either LPS or CpG where there was a reduction in intracellular bacteria compared to the infecting (*t* = 0) count (Figure 3(b)). Both MH-S and J774A.1 cells were able to clear all LVS when stimulated with LPS and IFN-*γ* in combination. The inhibition of bacterial growth appeared to correlate with the measurable levels of nitrite (Figures 2(a) and 2(b)).

*Intracellular growth of Schu S4* was less inhibited than LVS growth by the effects of the stimulants in either cell line and was unaffected by stimulation with either IFN-*γ* alone or CpG. In stimulated MH-S cells, Schu S4 growth was reduced by stimulation by LPS or LPS combined with IFN-*γ*, compared to growth in unstimulated cells (Figure 3(b);  *P* < 0.001). This occurred despite little or no measurable nitrite production. Stimulation of MH-S cells with IFN-*γ* plus LPS resulted in a significant reduction in Schu S4 from the starting infection with only 1 out of 3 experiments having detectable bacteria. By contrast Schu S4 growth in J774A.1 cells (Figure 3(a)) was largely unaffected by stimulation, despite levels of nitrite that had appeared to control LVS growth previously. The only exception to this was from stimulation with LPS and IFN-*γ* in combination, which significantly reduced intracellular Schu S4 (*P* < 0.05).

### 3.6. Effect of NO Inhibition

Stimulation with IFN-*γ*, LPS alone or in combination, or CpG alone in the presence of the nitric oxide synthase inhibitor (L-NMMA) of either cell line prevented nitrite production (concentrations consistently below 10 *μ*M, data not shown).

Compared with the levels of intracellular LVS seen in stimulated cells, stimulation combined with inhibition of nitric oxide synthase (NOS) resulted in an increase of intracellular LVS for most groups (Figure 3). In particular, the intracellular counts of LVS in stimulated CpG (both cell types) and stimulated LPS (MH-S only) were no longer significantly depressed compared to media only controls. However inhibition of NOS together with LPS and IFN-*γ* stimulation of MH-S-cells did not result in an increase in counts of intracellular LVS.

Schu S4 counts in L-NMMA-blocked and stimulated cells were almost unchanged by inhibiting nitrite production in either J774A.1 or MH-S cells. The only significant increase in growth occurred in the MH-S cells stimulated with LPS (*P* < 0.005) (Figure 3(b)). This was unexpected as nitrite production by MH-S cells stimulated with LPS in the presence of Schu S4 was not increased (Figure 2(b)). Despite inhibition of NOS in MH-S cells stimulated with LPS and IFN-*γ*, there was no increase in intracellular counts; there was a nonsignificant increase in the equivalent J774A.1 cells. This suggests that MH-S cells can control bacterial growth of either Schu S4 or LVS by additional pathway(s), possibly not present in J774A.1 cells.

## 4. Discussion

Initial infection studies illustrated that our strain of Schu S4 was significantly more infectious than our strain of LVS (although once infected the growth rate was comparable) and this was consistent not only for the macrophage cell lines reported here but also for epithelial cell lines such as A549 (personal observation). To our knowledge this has not been previously reported by others comparing these bacterial strains and is likely to be the result of our LVS being directly obtained from a vaccine vial and not passaged through animals as the ACTC strain is documented as being so.

### 4.1. Cell Differences Allow Greater Understanding of Intracellular Control

Here we have assessed the role of NO in the control of intracellular infections in two murine macrophage cell lines of different provenance: the alveolar MH-S cells and the peritoneal J774A.1 cells.

The two cell lines used in this study were deliberately selected based on their provenance. Due to their differing provenance, these cell lines might be expected to have different characteristics, through adaptation to their function* in vivo*, although continuous cell lines do not always retain the full characteristics of the primary cell [17]. In this study, we aimed to determine whether this difference in aetiology would affect the relative susceptibility of J774A.1 and MH-S cells to infection and the extent of subsequent intracellular growth of bacteria and to determine the role of NO production in limiting intracellular growth.

We have found a major difference between the cell lines in their resistance to infection with* Francisella* bacteria and our data indicate that MH-S cells are 10 times more resistant to infection than J774A.1 macrophages. In our studies, Schu S4 was consistently and significantly more rapidly phagocytosed in either cell line than the avirulent LVS, an observation not reported by others [18, 19]. However in agreement with these studies, once an infection was established, there was no difference in bacterial growth rate, between strains or cell types.

Stimulation of the cell lines to induce cytokine release revealed a similar cytokine profile; the only significant difference was increased TNF-*α* production in J774A.1 cells. In the absence of infection, nitrite levels in MH-S and J774A.1 cells varied with stimulant, in no particular pattern. The fact that J774A.1 cells failed to produce measurable increases in NO to infection under any of the test conditions is interesting. Previous studies have shown that concentrations of available arginine are crucial for production of NO [20]. Although there is more available arginine in RPMI used to maintain the MH-S cells than in DMEM used for the J774A.1 cells (Invitrogen) both media were supplemented with 10% foetal calf serum which increased the free arginine so there was no difference between culture conditions for the two cell lines. Others have used the same strategy; for example, clearance of* Burkholderia mallei* has been reported from cultured RAW macrophages in DMEM with 10% serum and attributed to the activation of NOS [21].

### 4.2. Nitric Oxide Is Important in Controlling LVS Infections But Not Schu S4

Infection of either cell type* in vitro* with either strain of* Francisella* did not induce nitrite production. This was expected since it is well documented that* Francisella *spp. possess a relatively inert form of LPS that fails to stimulate macrophages [22].

However stimulation of cells with a selection of native or synthetic bacterial products (LPS and CpG) alone or in combination with the proinflammatory IFN-*γ*, together with infection with LVS, resulted in enhanced nitrite production, which was significant for MH-S cells. The induction of nitrite levels in response to stimulation correlated with significant suppression of intracellular counts of LVS. Blocking NOS with L-NMMA resulted in increased intracellular counts in cells stimulated with LPS or CpG and this finding correlates well with previous reports [10, 14]. The fact that stimulated macrophages can produce TNF-*α* and consequently sufficient NO to prevent LVS replication has been reported before [1]; however, inhibition of J774A.1 cells occurs at high MOIs when the majority of macrophages are heavily infected [23].

In contrast to infection with LVS, infection of cells with Schu S4 in combination with stimulation caused no increase in nitrite production; stimulation of cells with IFN-*γ* or LPS was less protective against Schu S4 infection than against LVS infection. Interestingly, although CpG appeared to be a relatively poor stimulator of nitrite production, the inhibition of NOS was permissive for the intracellular growth of LVS as well as Schu S4, suggesting that CpG was not activating any other mechanisms of cellular resistance. This in part may explain why CpGs provide protection against lethal challenge of mice with LVS but are not able to protect against Schu S4 [24–26].

Few studies have explored Schu S4 growth in macrophages. Lindgren et al. [27] reported that cells stimulated with IFN-*γ* were more resistant to Schu S4 and also found that Schu S4 was more resistant than LVS to exposure to extracellular NO, something we were not able to demonstrate. Ireland et al. [28] found that pretreatment of cells with IFN-*γ* ensured sufficient NO activity to have a controlling effect on intracellular Schu S4 counts. We did not pretreat cells with stimulants, in order to avoid any effects on phagocytosis. However Ireland et al. [28] do note that posttreatment of cells with IFN-*γ* did not result in control of intracellular infections.

There is a significant problem with reactive nitrogen studies in the fact that measuring the stable end-product gives little information on the speed of generation of the reactive burst [29]. This is illustrated by our findings of control of Schu S4 numbers in MH-S cells stimulated with LPS and increased intracellular growth when NO production was blocked, despite any detectable increase in the overall production of nitrite.

### 4.3. Schu S4 Restricts NO Production

Schu S4 appears to have a mechanism, lacking in LVS, which prevents cellular nitrite production and this may be a significant factor in its virulence [16]. When macrophages generate NO, arginine is converted to citrulline, which can then be recycled by the cell to enable a sustained production of NO. Citrulline ureidase breaks down the citrulline, preventing further NO generation. Thus only in high concentrations of arginine can enough NO be generated to restrict Schu S4 growth. Interestingly although primarily isolated macrophages have been shown to limit intracellular growth of Schu S4 following stimulation with IFN-*γ*, these cells are not capable of producing NO [30]. Schu S4 appeared more able to restrict NO production in MH-S than in J774A.1 cells suggesting that J774A.1 cells hold more arginine intracellularly than MH-S cells.

This mechanism occurs over and above the activity of the superoxide dismutase that neutralises both reactive oxygen and nitrogen spp. [31] and is just one of many mechanisms employed by* F. tularensis* to manipulate and evade the host response [32].

### 4.4. Fully Stimulated Cells Do Not Rely upon NO

The LPS and IFN-*γ* mixed stimulus was able to fully inhibit bacterial growth for both Schu S4 and LVS in MH-S macrophages, despite the inhibition of cellular NOS. The combination of LPS and IFN-*γ* would be expected to have pleiotropic effects on macrophages in culture, apart from the induced nitrite secretion observed here for both cell lines. LPS and IFN-*γ* stimulation has also been reported to induce apoptosis [33]. This effect was not tested in our assay, but induction of apoptosis would significantly reduce intracellular bacterial counts.

In conclusion, NO production is a significant defence mechanism against bacterial infection in macrophages. Our results indicate that NO production in macrophage cell lines of different physiological provenance is sufficient to curtail the intracellular replication of LVS but not adequate on its own to control Schu S4. However, alveolar-derived MH-S macrophages were ten times more resistant to infection than J774A.1 cells, which are of peritoneal provenance. These data highlight both the importance of NO production to protect mammalian cells against intracellular infection and also the importance of choosing the cell line most appropriate to the route of infection to analyse host-pathogen interactions* in vitro*. In combination* in vitro*, LPS and IFN-*γ* are potent stimulators of mammalian cells and NO induction is a significant component in the cellular response. Here, we have demonstrated that such stimulation has resulted in a significant enhancement of the resistance to infection* in vitro*, even to the highly virulent Schu S4 strain.

Recent* in vitro* and* in vivo* studies have addressed the effects of LVS or Schu S4 infection on cytokine responses in a range of lung cells and more specifically alveolar macrophages at both the protein [34] and gene [35] levels, in the context of identifying responses which may correlate with protection. The current study has extended these data by demonstrating the importance of NO production by macrophages in resistance to infection. It further confirms the findings of Mahawar [16] by demonstrating that the differential infectivity of LVS and Schu S4 is partly due to differences in their capacity to restrict NO production by host cells. The fact that the virulent Schu S4 strain should express so many factors aimed at limiting reactive nitrogen serves to demonstrate what an effective protective cellular mechanism this is [32]. This work takes further steps towards understanding differences in mammalian cell responses to the virulent* Francisella* type A strain and LVS, an avirulent type B strain.

## Figures and Tables

**Table 1:** Comparison of *F*. *tularensis* colonisation of cell lines at 30 minutes of exposure at an MOI of 10 : 1 and 120 minutes at MOI of 1 : 1, measured in cfu/mL. Under either condition in either cell line Schu S4 infected in significantly higher numbers (*P* < 0.005 by Student's *t*-test). Values are the means from at least three independent experiments.

	30 min 10 : 1		120 min 1 : 1	
	LVS	Schu S4	LVS	Schu S4
J774A.1	1.53 × 10^2^	3.28 × 10^3^	9.30 × 10^2^	5.93 × 10^4^
(±SD)	4.16 × 10^1^	1.90 × 10^2^	1.44 × 10^2^	1.79 × 10^4^

MH-S	4.05 × 10^1^	1.03 × 10^2^	2.57 × 10^2^	8.00 × 10^3^
(±SD)	1.84 × 10^2^	3.86 × 10^1^	1.63 × 10^2^	1.21 × 10^3^

**Table 2:** Effect of increased concentration of nitric oxide on bacterial counts (cfu/mL) after 1 hr incubation in PBS.

Nitric oxide concentration (*μ*M)	Schu S4	LVS
0	3.23 × 10^4^	5.00 × 10^4^
0.125	1.90 × 10^4^	4.98 × 10^4^
0.25	1.55 × 10^4^	2.00 × 10^4^
0.5	8.75 × 10^3^	9.00 × 10^3^
1	6.25 × 10^3^	9.97 × 10^3^
2	2.75 × 10^3^	6.00 × 10^3^
